# Efficacy and Safety of Intravenous rtPA in Ischemic Strokes Due to Small-Vessel Occlusion: Systematic Review and Meta-Analysis

**DOI:** 10.1007/s12975-021-00890-9

**Published:** 2021-02-28

**Authors:** Bartosz Karaszewski, Adam Wyszomirski, Bartosz Jabłoński, David J. Werring, Dominika Tomaka

**Affiliations:** 1 Department of Adult Neurology, Division of Neurology, Faculty of Medicine, Medical University of Gdansk, Poland, Gdansk, Poland; 2 Department of Adult Neurology, University Clinical Center in Gdansk, Poland, Debinki 7, 80-211 Gdansk, Poland; 3 Main Expert in Stroke Medicine for the Polish Ministry of Health, Warsaw, Poland; 4 Stroke Research Centre, University College London, Queen Square Institute of Neurology, London, UK

**Keywords:** Stroke, Small vessel disease, Lacunar infarction, Brain ischemia, Thrombolysis, Meta-analysis

## Abstract

Intravenous recombinant tissue plasminogen activator (iv-rtPA) has been routinely used to treat ischemic stroke for 25 years, following large clinical trials. However, there are few prospective studies on the efficacy and safety of this therapy in strokes attributed to cerebral small vessel disease (SVD). We evaluated functional outcome (modified Rankin scale, mRS) and symptomatic intracerebral hemorrhage (sICH) using all available data on the effects of iv-rtPA in SVD-related ischemic stroke (defined either using neuroimaging, clinical features, or both). Using fixed-effect and random-effects models, we calculated the pooled effect estimates with regard to excellent and favorable outcomes (mRS=0–1 and 0–2 respectively, at 3 months), and the rate of sICH. Twenty-three studies fulfilled the eligibility criteria, 11 of which were comparative, and there were only 3 randomized clinical trials. In adjusted analyses, there was an increased odds of excellent outcome (adjusted OR=1.53, 95% CI: 1.29–1.82, I2: 0%) or favorable outcome (adjusted OR=1.68, 95% CI: 1.31–2.15,I2: 0%) in patients who received iv-rtPA compared with placebo. Across the six studies which reported it, the incidence of sICH was higher in the treatment group (M-H RR = 8.83, 95% CI: 2.76–28.27). The pooled rate of sICH in patients with SVD administered iv-rtPA was only 0.72% (95% CI: 0.12%–1.64%). We conclude that when ischemic stroke attributed to SVD is considered separately, available data on the effects of iv-rtPA therapy are insufficient for the highest level of recommendation, but it seems to be safe. Although further therapeutic trials in SVD-related ischemic stroke appear to be justified, our findings should not prevent its continued use for this group of patients in clinical practice.

**Supplementary Information:**

The online version contains supplementary material available at 10.1007/s12975-021-00890-9.

## Background

Intravenous administration of recombinant tissue plasminogen activator (rtPA) has been a standard treatment for acute ischemic stokes for 25 years, based on the results of large clinical trials [1–6]. However, these studies recruited patients regardless of the size and type of the occluded artery, and post hoc analyses in small vessel occlusion attributed to cerebral small vessel disease (SVD) have been limited.

The only major published randomized subgroup analyses on SVD-related strokes are from the Third International Stroke (IST-3) and The *National Institute of Neurological Disorders and Stroke (*NINDS) trials [6, 7]. In the former, the largest, “lacunar stroke (LS)” was diagnosed in 168 (11.09%) of 1515 IVT-treated patients. Symptomatic intracranial hemorrhage (sICH; defined as “clinically significant deterioration or death within the first 7 days of treatment with evidence of either significant brain parenchymal hemorrhage or significant hemorrhagic transformation on brain imaging”) occurred in 4.76% (8/168) of LS-patients, compared with 7.13% (96/1347) in the non-LS-group. The adjusted effect of treatment on the primary outcome (alive and independent, Oxford Handicap Score 0, 1, or 2; OHS 0–2 versus 3–6 measured at 6 months) was 100/168 (59.5%) in IVT-treated patients versus 103/164 (62.8%) in non-IVT-treated; OR 0.91 (95% CI: 0.48–1.72). In the NINDS cohort, fifty-one (16.34%) of IVT-treated patients had SVD stroke with a favorable functional outcome on the modified Rankin scale in 32 (63.0%). However, no data on the sICH rates in this patient subgroup have been reported. The ATLANTIS, ECASS, and ECASS-3 research groups [1, 3, 5] have not reported specific data concerning the LS/SVD patients. In a post hoc analysis of the WAKE-UP [8] trial, in patients with LS, a favorable outcome was observed in 59% (31 of 53) of i.v. rtPA -treated patients compared with 46% (24 of 52) of those non-thrombolysed (OR 1.67 [95% CI: 0.77–3.64]). However, the WAKE-UP trial rtPA administration criteria were different to those of standard use (i.e. known time of stroke onset, CT as the primary patient selection neuroimaging tool). Moreover, the subgroup of patients in WAKE-UP with LS made up only 21.5% of all patients (108 of 503; most patients presented with mild to moderate deficits), and the trial was not powered to demonstrate the efficacy of treatment in stroke subgroups [9].

Strokes caused by acute occlusion of small perforating arterioles of the brain have different etiological, pathophysiological, and clinical characteristics to those resulting from occlusion attributed to large-vessel disease (LVD) (Table 1) [10–13]. For example, in many patients with small vessel occlusion, brain imaging shows established SVD (e.g., white matter lesions, multiple chronic lacunar infarctions, or cerebral microbleeds, potentially increasing rtPA-related sICH risk) [14]. We hypothesized that the benefits and harms of reperfusion therapy with rtPA might be different in patients with small-vessel occlusion compared with those with large-vessel occlusion, with implications for optimal stroke management and future trials.

**Table 1:** Specific features of strokes caused by the occlusion of small vessels

- Affects single, long, and mostly unbranched small deep perforating or lenticulostriate arteries or arterioles, usually about 100 to 400 μm in diameter	
- Strokes are attributable to small infarcts < 1.5 cm in diameter, usually in the basal ganglia, internal capsule, thalamus, corona radiata, or brainstem	
- Differing histological structure of the wall of occluded vessels (capillaries are characterized by a single layer of highly specialized endothelial cells with extensive tight junctions (no fenestrations) and pericytes on the basal lamina, without smooth muscle; parenchymal arterioles, unlike pial arteries and arterioles, are in direct contact with astrocytes and neuronal tissue)	
- Disparate specific paracrine function of endothelial cells of small vessels, including the regulation of the production and release of tPA	
- Different underlying mechanisms of ischaemia (including reduced arterial flow from a pathological process related to blood brain barrier disruption, endothelial dysfunction, etc. rather than in situ thrombosis or thrombo-embolism from extracranial arteries or the heart)	

In this systematic review, we combined all available data on the effects of rtPA in subgroups of patients with acute ischemic stroke attributed to small vessel occlusion, including large clinical trials and other observational studies.

We compared the effects of i.v. rtPA versus placebo on functional outcome based on the modified Rankin Scale (mRS) at 90 days from stroke onset as a primary endpoint, the only available outcome measure in most of these studies. We did separate meta-analyses for mRS 0–1 (excellent outcome) and 0–2 (favorable outcome). As a safety measure, we analyzed the rate of rtPA-related sICH in stroke attributed to small vessel occlusion across all studies.

## Materials and Methods

### Search Strategy

This study was conducted per the Preferred Reporting Items for Systematic Reviews and Meta-Analyses (PRISMA) [15]. Two of the authors (BJ and DT) systematically reviewed the MEDLINE (PubMed) and Scopus bibliographic databases for the appropriate articles that were published before the end of May 2019. The following search strategy was applied: (“lacunar”[Title/Abstract] OR “small vessel”[Title/Abstract] OR “small artery”[Title/Abstract] OR “minor stroke”[Title/Abstract]) AND (“thrombolysis”[Title] OR “rtPA”[Title] OR “actilyse”[Title] OR “alteplase”[Title] OR “fibrinolysis”[Title]) for MEDLINE, and similar for Scopus (Supplemental Fig. 1).

### Inclusion and Exclusion Criteria

All papers were examined independently with regard to the inclusion and exclusion criteria by 2 authors (BJ and DT), according to the PICOS structure (participants, intervention, comparison, outcome, and study design) [15]. The investigators selected papers that met each of the following criteria:

1. Studies recording the effects of i.v. rtPA in adult patients with SVD-associated acute ischemic stroke defined using clinical or radiological criteria (or both) in observational studies and controlled trials, regardless of design.
2. Sub-classification of acute ischemic stroke including small vessel occlusion (lacunar), as per the Trial of Org 10,172 in Acute Stroke Treatment (TOAST) or Oxfordshire Community Stroke Project (OCSP).
3. Functional outcome assessed using the modified Rankin Scale (mRS) [16] at 3 months and/or safety measured by the rate symptomatic intracerebral hemorrhage (sICH).

The investigators screened all reference lists to identify other relevant studies. Disagreements between reviewers were resolved by mutual consensus with final adjudication when required by the senior author (BK).

We excluded case studies, reviews, meta-analyses, editorials, letters to the editor, commentaries, abstracts from conference proceedings, and papers not published in English.

### Outcomes

We defined functional outcome as (1) excellent, defined as mRS from 0 to 1 at 3 months. and (2) favorable, defined as an mRS of ≤ 2 at 3 months. For these outcomes, we included only controlled clinical trials. We assessed symptomatic intracerebral hemorrhage (sICH) using a standardized definition (NINDS tPA trials, the European-Australian Cooperative Acute Stroke Study 2 (ECASS2), or a modified version of the Safe Implementation of Thrombolysis in Stroke Monitoring Study (mSITS-MOST)), extracted from both randomized controlled and observational studies (the latter including comparative: rtPA vs placebo but not randomized, and non-comparative, i.e., with no placebo).

### Sensitivity Analysis

We did not have data as to whether patients in the studies in our meta-analysis were simultaneously registered into the SITS-MOST database. To reduce bias, we performed a sensitivity analysis excluding SITS-MOST data reported by Matusevicius et al.

### Risk of Bias Assessment

The Cochrane Risk of Bias tool [17] was used to evaluate the recruited studies (randomized controlled trials). The criteria covered the following items: selection bias (random generation sequence and allocation concealment), performance bias (blinding of participants and personnel), detection bias (blinding of outcome assessment), attribution bias (incomplete outcome data), and reporting bias (selective reporting). Each item was classified as low, unclear, or high risk of bias.

The Newcastle-Ottawa Scale (NOS) tool [18] was applied to explore the potential sources of bias amongst the included observational comparative studies. The approach based on NOS consists of 9 items divided into three areas: the selection of studies, the comparability of studies, and the assessment of exposure/outcome. Each study might be assessed on up to nine points. A score of 6 or more is considered to indicate the satisfactory quality of the study. The assessment was performed independently by three reviewers (AW, DT, and BJ), and any discrepancies were resolved in a group investigator discussion.

### Statistical Analysis

We applied the DerSimonian and Laird random-effects model to estimate the risk ratios (RRs) for excellent and favorable functional outcomes for the unadjusted analysis. We used the fixed-effect model to estimate adjusted odds ratios (aORs) based on multivariable logistic regression data for the adjusted analysis.

We performed this dual approach analysis (non-adjusted/adjusted) due to important limitations of each, combined with data availability (trials, observational studies). The former (non-adjusted) ignores any clinical differences (baseline risk factors) between the examined populations other than the acute stroke although it was applied in majority of other papers including the work of Matusevicius et al. The adjusted analysis suffers from the high heterogeneity of the original studies, especially those non-randomized, including different baseline risk factor characteristics, and consists of only a few randomized placebo-controlled trials and three comparative observational studies. Furthermore, importantly, the adjusted odds ratios showed in primary studies overestimated the positive effect [19].

In the comparative studies where sICH events were rare or absent, we used the Mantel-Haenszel risk ratio (M-H RR) under the fixed-effect model. The incidence of sICH for noncomparative studies was expressed as a percentage, and the results were calculated using the random-effects model by applying Freeman-Tukey double arcsine transformation. The I^2^ statistic was used to evaluate heterogeneity across the studies for all meta-analyses. The effect size estimates were reported using 95% confidence intervals (CIs). The possibility of publication bias was assessed by visual analysis of funnel plot. All statistical analyses were conducted using the “meta” package in R (version 3.6.1).

## Results

Our literature search identified 229 records from the MEDLINE and Scopus databases. Forty-two articles underwent a full-text evaluation, 19 of which were excluded, leaving altogether 23 studies in this systematic review [6–8, 20–39]. The selection process is reported in a PRISMA flowchart (Supplemental Fig. 1). We identified 3 RCTs and 8 comparative non-randomized trials; the remaining studies were observational and noncomparative (Tables 2 and 3). SVD/LS and sICH definitions used in each of the selected studies are listed in Supplemental Table 1.

**Table 2:** Table characteristics of comparative (randomized and non-randomized) studies

Reference	Study period	Study design	Number of patients with LACI	Stroke classification	Median NIHSS improvement^#^	% of mRS 0–1	% of mRS 0–2	% of sICH	% of mortality
			rtPA/ control		rtPA/ control				
Griebe et al. [21]	2004–2011	Observational; retrospective; single-center	69/ 468	TOAST	3/ 1	46/ 49	63/ 74	0/ 0	0/ 0
Eggers et al. [22]	2003-	Observational; retrospective; multicenter	401/ 401	OCSP TOAST	3/ 2	69/ 52	80/ 69	1/ 0.25	4/ 3
Hwang et al. [23]	2006–2007	Observational; retrospective; single-center	29/ 47	OCSP	–	79/ 81	–	–	–
Lahoti et al. [24]	5 years	Observational; retrospective; multicenter	54/ 48	TOAST	–	65/ 63	–	3.7/ 0	–
NINDS Group [6]	1991–1994	RCT; multicenter	51/ 30	TOAST	–	63/ 40	–	–	–
Shobha et al. [25]	2003–2008	Case-cohort; retospective; multicenter	195/ 2001	OCSP	–	–	1.25 (1.08–1.44)*	1.5/ -	–
Lindley et al. [7]	2000–2012	RCT; multicenter	168/ 164	OCSP	–	–	–	4.8/ 0	–
Barow et al. [8]	2012–2017	RCT; multicenter	55/ 53	TOAST	–	59/ 46	87/ 79	1.8/ 0	2/ 2
Paek et al. [26]	2011–2016	Observational; retrospective; multicenter	194/ 2289	TOAST	–	57/ 67	81/ 85	1.6/ 0.04	0/ 0.7
Yang et al. [27]	2008–2015	Observational; retrospective; single-center	57/ 27	TOAST	-2 / 0^$^	64/66	–	–	–
Matusevicius et al. [20]	2002–2016	Observational; retrospective; multicenter	4610/ 1221	TOAST	–	65/ 63	82/ 81	0.4/ -	2/ 2.6

mRS: modified Rankin Scale; RR: risk ratio; CI: confidence interval; rtPA: recombinant tissue plasminogen activator; RCT: randomized controlled trial; sICH: symptomatic intracerebral hemorrhage; LACI: lacunar infarct; TOAST: The Trial of Org 10,172 in Acute Stroke Treatment; OCSP: The Oxfordshire Community Stroke Project; *: crude RR (95% CI); #: Δ_initial-discharge_; $: 24 h-NIHSS-shift

**Table 3:** Table characteristics of non-comparative observational (no placebo) studies

Reference	Study period	Study design	Number of rtPA patients with LACI	Stroke classification	Median NIHSS improvement^#^	% of mRS 0-1	% of mRS 0–2	% of sICH	% of mortality
Chang et al. [28]	2007–2010	Observational; retrospective; single-center	39	TOAST	1.6^$^	–	–	4	–
Sung et al. [29]	2003–2012	Observational; retrospective; single-center	22	OCSP	–	–	81.8	4.5	0
Lee et al. [30]	1999–2008	Observational; retrospective; single-center	23	OCSP	–	–	–	0	–
Fluri et al. [31]	−2007	Observational; retrospective; multicenter	65	TOAST	–	–	75.4	4.6	1.5
Kohrmann et al. [32]	2006–2008	Observational; retrospective; single-center	12	TOAST	–	–	–	0	–
Padma et al. [33]	2002–2006	Observational; prospective; single-center	22	TOAST	–	–	–	0	–
Cocho et al. [34]	1997–2004	Observational; retrospective; single-center	11	OCSP	–	27.2	54.5	0	0
Miedema et al. [35]	2002–2013	Observational; retrospective; multicenter	162	OCSP	–	–	78	1.9	–
Simonsen et al. [36]	2004–2010	Observational; retrospective; single-center	115	TOAST	–	–	–	0	–
Pan et al. [37]	2007–2014	Observational; retrospective; multicenter	82	TOAST	–	–	–	1.2	0
Zivanovic et al. [38]	2009–2016	Observational; prospective; single-center	46	OCSP	–	76.1	82.6	0	2.2
Kim et al. [39]	2010–2016	Observational; retrospective; single-center	25	TOAST	–	52	–	0	–

rtPA: recombinant tissue plasminogen activator: mRS: modified Rankin Scale; sICH: symptomatic intracerebral hemorrhage; LACI: lacunar infarct; TOAST: The Trial of Org 10,172 in Acute Stroke Treatment; OCSP: The Oxfordshire Community Stroke Project; #: Δ_initial-discharge_; $: mean difference in discharge NIHSS

### Risk of Bias Assessment

Retrospective reports accounted for 18/23 (78%) of all selected studies. The methodological quality of the randomized controlled trials is presented in Supplemental Table 2. There is low level of bias within the following items: selection bias, detection bias, and reporting bias. The high risk of bias in the blinding of participants and personnel section was found in one study, whereas another one had incomplete outcome data. Results of the investigation towards risks of bias across the observational studies are demonstrated in Supplemental Table 3 revealing no major problems.

Although there is no evident publication bias in the visual analysis of the funnel plots of the pooled unadjusted estimates (favorable outcome, excellent outcome, incidence of sICH in rtPA vs control patients; Supplemental Fig. 2a-c), too small number of points/studies excludes any robust assessment. The only potential publication bias might have been caused by high variation between results of larger studies in the analysis of the pooled unadjusted estimate for sICH in thrombolyzed patients (Supplemental Fig. 2d).

### Outcomes

**Fig. 1:**
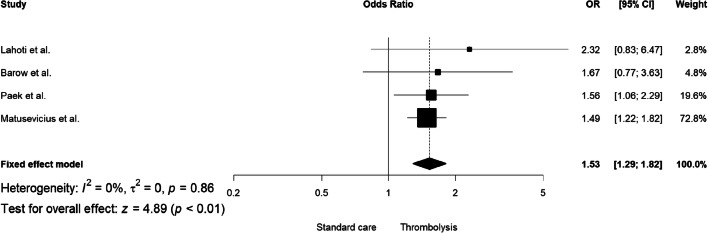
The adjusted pooled odds ratio for excellent functional outcome

**Fig. 2:**
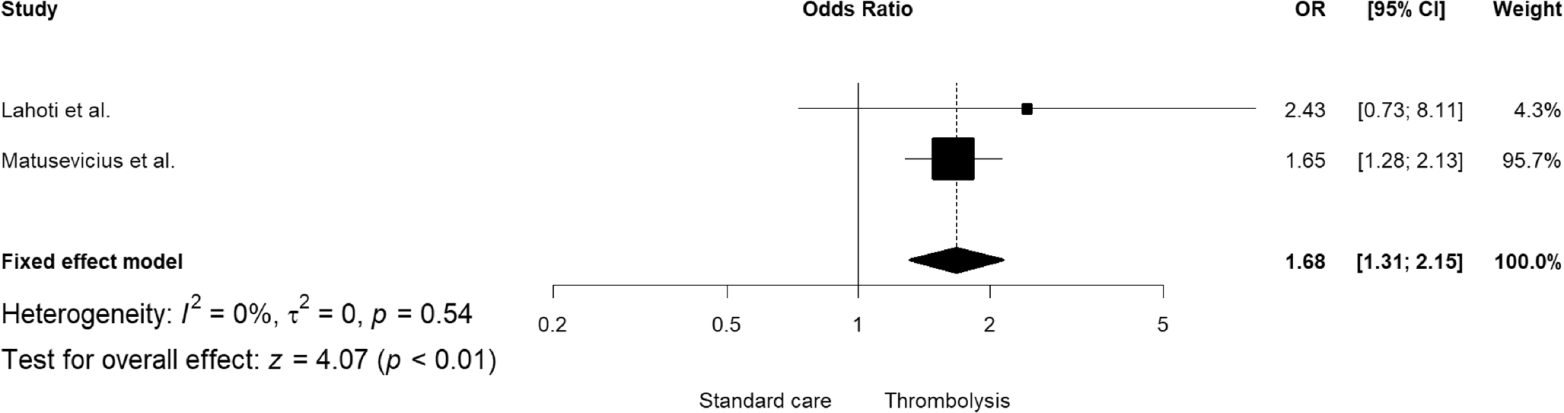
The adjusted overall odds ratio for favorable functional outcome

In the analysis of unadjusted effect, there was no significant association between tPA (compared to placebo) and excellent functional outcome (RR = 1.05, 95% CI: 0.93–1.18, I^2^: 74%; Supplemental Fig. 3) or favorable functional outcome (RR = 1.06, 95% CI: 0.97–1.15, I^2^: 81%; Supplemental Fig. 4) in patients with SVD-associated ischemic stroke. However, the adjusted analysis showed an association of rtPA with both excellent and favorable functional outcomes was positive (adjusted odds ratio aOR = 1.53, 95% CI: 1.29–1.82, I^2^: 0%; Fig. 1 and aOR = 1.68, 95% CI: 1.31–2.15, I^2^: 0%; Fig. 2, respectively). Only four comparative studies (36.4%) reported the analysis of excellent and favorable outcomes after adjustment for covariates. For three observational and comparative studies adjustment included basic demographic risk factors (age, sex) and NIHSS at baseline. Further information about adjustment for covariates is presented in Supplemental Table 4.

**Fig. 3:**
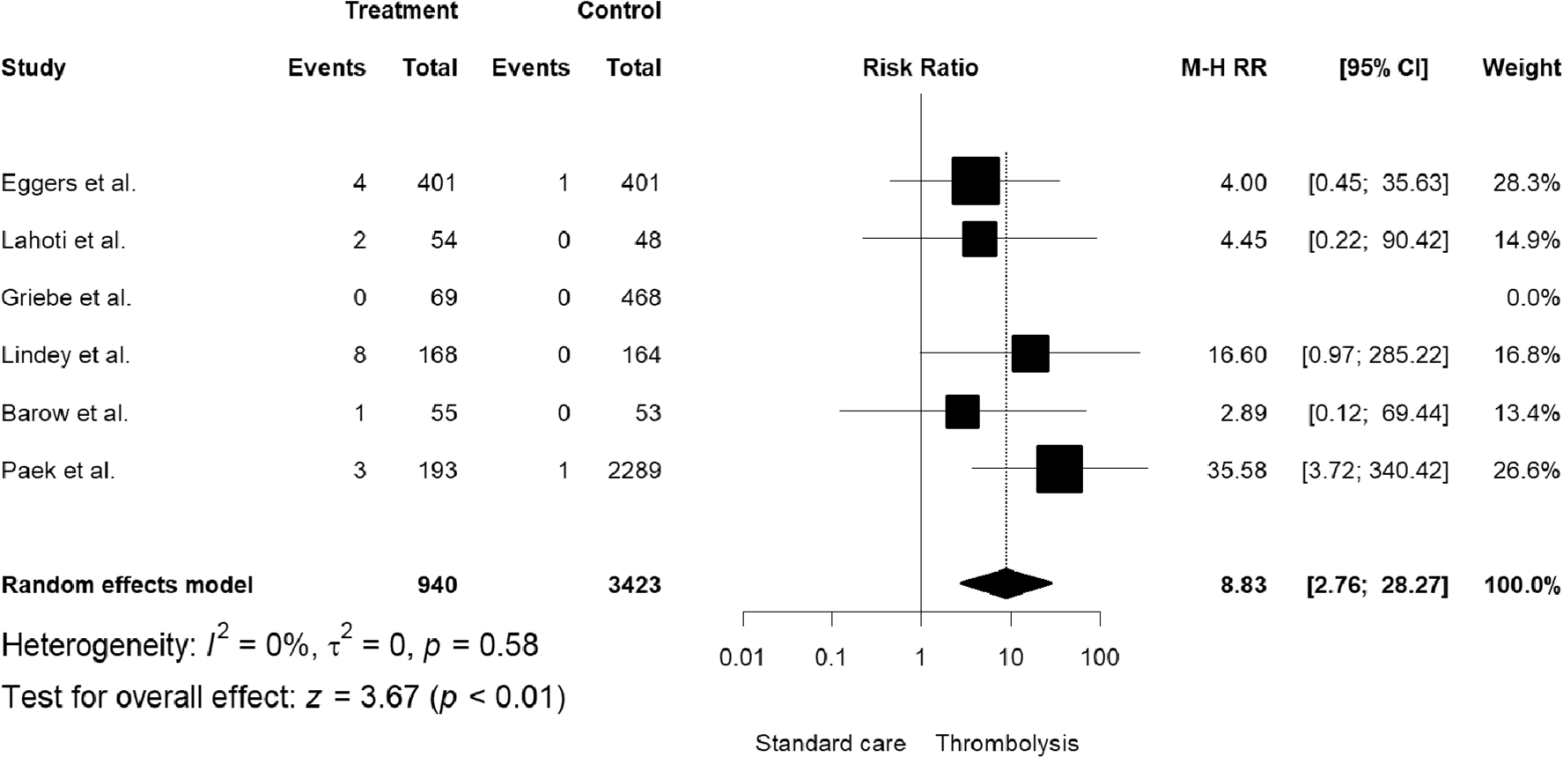
The incidence of sICH expressed as the Mantel-Haenszel risk ratio

**Fig. 4:**
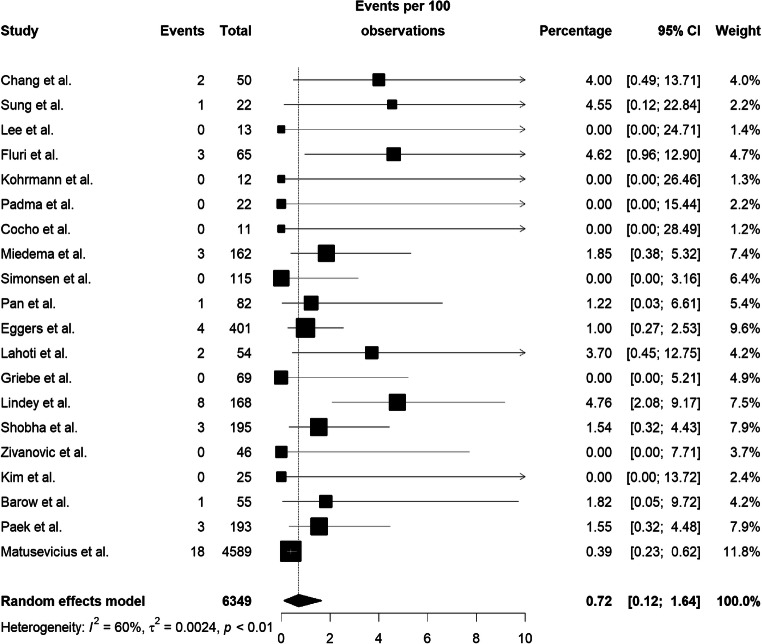
The pooled rate of sICH in trombolysed patients

The incidence of sICH was significantly higher in thrombolyzed patients (compared with placebo; M-H RR = 8.83, 95% CI: 2.76–28.27, I^2^: 0%; Fig. 3; Table 2). The pooled estimate of the rate of sICH in patients who were treated with alteplase was 0.72% (95% CI: 0.12%–1.64%, I^2^: 60%; Fig. 4; Table 3); this is much lower than the rate observed in major and inclusive stroke thrombolysis trials (18/940 versus 2/3423 events in thrombolyzed or non-thrombolyzed, respectively).

### Sensitivity Analysis

We obtained similar findings in sensitivity analyses excluding a recent SITS-MOST publication (in the case of possible data duplication). The overall effects of both excellent and favorable functional outcomes in the unadjusted meta-analysis that excluded SITS-MOST registry were insignificant (RR = 1.06, 95% CI: 0.90–1.26, I^2^: 77%; Supplemental Fig. 5, RR = 1.07, 95% CI: 0.93–1.22, I^2^: 84%; Supplemental Fig. 6, respectively), whereas the adjusted pooled effect for the excellent outcome was significant (aOR = 1.64, 95% CI: 1.19–2.28, I^2^: 0%; Supplemental Fig. 7). The pooled estimate of the rate of sICH in patients who were treated with alteplase excluding those from SITS-MOST registry was 0.82% (95% CI: 0.28%–1.53%, I^2^: 10%; Supplemental Fig. 8).

## Discussion

Treatment of ischemic strokes that are caused by the occlusion of small vessels with rtPA up to 3–4.5 h from the initial symptoms is included in most national stroke therapeutic guidelines as a strong recommendation. This recommendation is based on large randomized studies that have tested the efficacy of alteplase in acute cerebral ischemia of any subtype with an assumption that the effects of the treatment of all of these stroke subtypes are similar (or with limited post hoc subgroup analyses).

Cerebral SVD is associated with pathologies in perforating cerebral arterioles, capillaries, and venules, with characteristic although not specific imaging findings such as white matter hyperintensities, lacunes, microbleeds, visible perivascular spaces, atrophy, and lacunar strokes (STRIVE, STandards for Reporting and Imaging of Small Vessel Disease [40]). SVD is responsible for up to 45% of dementias and about 20% of all acute strokes [41]. By contrast, large-vessel disease (LVD) accounts for approximately 20–30% of all ischemic strokes and refers to various arterial pathologies including extracranial or intracranial atherosclerosis. Unfortunately, there is little empirical information on the effects of rtPA in SVD versus in LVD ischemic strokes. In the NINDS cohort, fifty-one (16.34%) of IVT-treated patients had SVD stroke, with a favorable functional outcome (mRS) in 63.0% of patients, compared with 40.0% in large artery atherosclerosis (LAA). SVD stroke patients had also a better functional outcome prognosis versus LAA according to Helsinki Thrombolysis Stroke Registry [42] (excellent outcome in 57 vs 31%, with sICH (ECASS criteria) in 0.0 vs 7.4%).

Our findings in an unadjusted meta-analysis of the available data did not show benefit from tPA in SVD-associated acute ischemic stroke; thus, the assumption that small vessel occlusions behave in the same way as other stroke subtypes might not be correct. That strokes due to small vessel occlusion might have a different response to tPA is plausible because of the fundamental differences in the underlying disease processes causing large- and small-vessel occlusions (Table 1). On the other hand, adjusted analysis revealed that SVD/LS patients who received i.v. rtPA might have an increase in the odds of a better outcome than those receiving placebo or no thrombolytic treatment. There are no dedicated randomized trial data on the effects of rtPA in SVD-related ischemic stroke. Thus, on the basis that ischemic stroke related to small vessel occlusion (SVD ischemic stroke) is considered as a distinct stroke subtype, we suggest that current data are insufficient to recommend iv-rtPA treatment based on Level 1 or Level 2 evidence; rather, it instead fulfills Level 3 criteria of Oxford Centre for Evidence-Based Medicine within the “Treatment Benefits” assessment (outcome of adjusted or unadjusted analysis respectively) [43]. In designing this study, it was also important for us to be mindful of the potential risks of sICH because the expected outcome of SVD-associated ischemic stroke is favorable in as many as 66–79% [44, 45] without specific medical care. Our review of observational studies suggested that the rate of symptomatic ICH is higher in those who are treated with rtPA than those who are not. However, the absolute rate of sICH, although higher than in those not treated, is still low (pooled estimate 0.72%), much lower than the rate of 3–6% observed in the large inclusive stroke thrombolysis trials. This all supports the continued use of tPA in SVD-associated ischemic stroke because—although the data only fulfill criteria for lower evidence strength and recommendation for use—it seems to be reasonably safe. Similar considerations were the major issue of the PRISMS [46] trial—although it concerned minor acute ischemic strokes with deficits scored as 0–5 on the NIHSS and judged as not disabling, of which as many as 32% met the criteria to be SVD strokes. The PRISMS group, however, did not report mRS results for the extracted SVD-associated stroke subpopulation.

The mRS sensitivity in distinguishing minor functional improvements and deterioration, including those related to key non-motor domains, is low, which must be taken into account specifically when analysing outcomes of strokes attributed to SVD, and thus in interpreting results of this meta-analysis [47, 48]. Furthermore, a major limitation of the adjusted analysis is associated with substantial differences in selected confounders between the studies. These discrepancies in selection and accounting for risk factors might have obviously caused overestimation or underestimation of the outcome effect due to the non-collapsibility of the odds ratio [49].

Shortly before we finished working on this meta-analysis, Matusevicius et al. published a report with partially similar aims. There are however many differences in the design, contents, and composition between these two works. Most importantly, unlike Matusevicius et al., we included only comparative studies (rtPA treated SVD / LS patients versus those not treated or placebo-treated), and did not compare effects of rtPA between SVD/LS versus non-SVD/LS stroke patients. Instead, we compared the therapeutic effects for rtPA versus no or placebo treatment separately for favorable and excellent outcomes (mRS 0–2 and 0–1 respectively) and furthermore calculated the risks of rtPA-associated sICH in SVD stroke patients and expanded the rtPA—placebo efficiency comparison by an adjusted meta-analysis [8, 20, 24, 26]. Finally, it is worth noting that our work was designed and strictly based on the PRISMA protocol [15].

In conclusion, if treated as a separate disorder, the current empiric data on the effects of i.v. rtPA in SVD-related ischaemic stroke are insufficient to provide high-level evidence-based recommendations. Further sub-analysis of past thrombolysis trials is unlikely to resolve this issue, mainly because of limitations in their design, including outcome measures that might be insensitive to the consequences of small vessel occlusions, and the need for post hoc analyses. Moreover, in the acute clinical setting, the diagnosis of whether the stroke is of SVD origin is not always straightforward. In the OCSP classification (The Oxfordshire Community Stroke Project classification) patient cohort, the sensitivity and specificity of LS diagnosis were 70 and 93%, respectively [50].

Although our findings do not suggest any change in current rtPA protocols for SVD-associated ischemic stroke, we identified a lack of high-quality evidence in this stroke subgroup. We suggest that prospective, randomized controlled trials of thrombolysis or other novel interventions specifically targeting acute small vessel occlusion are needed to provide high-level evidence of treatment efficacy. We suggest that for acute patient recruitment, such studies might need to implement clinical and radiological criteria (using diffusion-weighted MRI) to diagnose lacunar stroke rather than acute non-contrast CT neuroimaging, because of the limited sensitivity of the latter in the acute phase.

## Supplementary Information

ESM 1 (DOCX 1.44 MB)

## Data Availability

Not applicable.

## References

[1] Hacke W, Kaste M, Cesare F, Toni D, Lesaffre E, von Kummer R (1995). Intravenous thrombolysis with recombinant tissue plasminogen activator for acute hemispheric stroke. JAMA.

[2] Hacke W, Kaste M, Fieschi C, von Kummer R, Davalos A, Meier D, Larrue V, Bluhmki E, Davis S, Donnan G, Schneider D, Diez-Tejedor E, Trouillas P (1998). Randomised double-blind placebo-controlled trial of thrombolytic therapy with intravenous alteplase in acute ischaemic stroke (ECASS II). Lancet.

[3] Hacke W, Kaste M, Bluhmki E, Brozman M, Dávalos A, Guidetti D (2008). Thrombolysis with Alteplase 3 to 4.5 hours after acute ischemic stroke Werner. N Engl J Med.

[4] Sandercock P, Wardlaw JM, Lindley RI, Dennis M, Cohen G, Murray G (2012). The benefits and harms of intravenous thrombolysis with recombinant tissue plasminogen activator within 6 h of acute ischaemic stroke (the third international stroke trial [IST-3]): a randomised controlled trial. Lancet.

[5] Clark WM, Wissman S, Albers GW, Jhamandas JH, Madden KP, Hamilton S (2003). Recombinant tissue-type plasminogen activator (Alteplase) for ischemic stroke 3 to 5 hours after symptom onset. JAMA.

[6] The National Institute of Neurological Disorders and Stroke rt-PA Stroke Study Group (1995). Tissue plasminogen activator for acute ischemic stroke. N Engl J Med.

[7] Lindley RI, Wardlaw JM, Whiteley WN, Cohen G, Blackwell L, Murray GD, Sandercock PAG, Baigent C, Chadwick D, Tyrrell P, Lowe G, Dennis M, Innes K, Goodare H, Farrall A, von Kummer R, Cala L, von Heijne A, Morris Z, Adami A, Peeters A, Potter G, Brady N, Collins R, Bath P, van Gijn J, Gray R, Hart R, Yusuf S, Muir K, Hankey GJ, Matz K, Brainin M, Peters A, Gubitz G, Phillips S, Ricci S, Arauz A, Berge E, Bruins Slot K, Czlonkowska A, Kobayashi A, Correia M, Lyrer P, Engelter S, Murray V, Norrving B, Terént A, Wester P, Venables G, Innes K, Clark A, Perry D, Soosay V, Buchanan D, Grant S, Sakka E, Drever J, Walker P, Herath I, Brown AL, Chmielnik P, Armit C, Walton A, Hautvast M, Lewis S, Heron G, Odusanya S, Linksted P, Kane I, Sellar R, White P, Keston P, Farrell A, Morris Z, Miranda H, Celani MG, Righetti E, Cenciarelli S, Mazzoli T, Cantisani TA, Bembenek J, Isaakson E, Trial Steering Committee, CT and MR reading panel, Data Monitoring Committee, Event Adjudication Committee, National Coordinators and Associate National Coordinators (2015). Alteplase for acute ischemic stroke: outcomes by clinically important subgroups in the third international stroke trial. Stroke.

[8] Barow E, Boutitie F, Cheng B, Cho TH, Ebinger M, Endres M, et al. Functional outcome of intravenous thrombolysis in patients with lacunar infarcts in the WAKE-UP trial. JAMA Neurol. 2019:1–9. 10.1001/jamaneurol.2019.0351 PMC6563546 30907934

[9] Thomalla G, Simonsen CZ, Boutitie F, Andersen G, Berthezene Y, Cheng B, Cheripelli B, Cho TH, Fazekas F, Fiehler J, Ford I, Galinovic I, Gellissen S, Golsari A, Gregori J, Günther M, Guibernau J, Häusler KG, Hennerici M, Kemmling A, Marstrand J, Modrau B, Neeb L, Perez de la Ossa N, Puig J, Ringleb P, Roy P, Scheel E, Schonewille W, Serena J, Sunaert S, Villringer K, Wouters A, Thijs V, Ebinger M, Endres M, Fiebach JB, Lemmens R, Muir KW, Nighoghossian N, Pedraza S, Gerloff C (2018). MRI-guided thrombolysis for stroke with unknown time of onset. N Engl J Med.

[10] Poggesi A, Pasi M, Pescini F, Pantoni L, Inzitari D (2016). Circulating biologic markers of endothelial dysfunction in cerebral small vessel disease: a review. J Cereb Blood Flow Metab.

[11] Wardlaw JM, Smith C, Dichgans M (2013). Mechanisms of sporadic cerebral small vessel disease: insights from neuroimaging. Lancet Neurol.

[12] Pantoni L (2010). Cerebral small vessel disease: from pathogenesis and clinical characteristics to therapeutic challenges. Lancet Neurol.

[13] Arboix A, Martí-Vilaita JL (2009). Lacunar stroke. Expert Rev Neurother.

[14] Charidimou A, Turc G, Oppenheim G, Yan S, Scheitz JF, Erdur H (2017). Microbleeds, cerebral hemorrhage, and functional outcome after stroke thrombolysis: individual patient data meta-analysis. Stroke.

[15] Liberati A, Altman DG, Tetzlaff J, Mulrow C, Gøtzsche PC, Ioannidis JPA, Clarke M, Devereaux PJ, Kleijnen J, Moher D (2009). The PRISMA statement for reporting systematic reviews and meta-analyses of studies that evaluate health care interventions: explanation and elaboration. J Clin Epidemiol.

[16] Rankin J (1957). Cerebral vascular accidents in patients over the age of 60: II. Prognosis. Scott Med J.

[17] Higgins JPT, Altman DG, Gotzsche PC, Juni P, Moher D, Oxman AD, Savovic J, Schulz KF, Weeks L, Sterne JAC, Cochrane Bias Methods Group, Cochrane Statistical Methods Group (2011). The Cochrane Collaboration’s tool for assessing risk of bias in randomised trials. BMJ.

[18] Wells GA, Shea B, O’Connell D, Peterson J, Welch V, Losos M, et al. The Newcastle-Ottawa Scale (NOS) for assessing the quality if nonrandomized studies in meta-analyses http://www.ohri.ca/programs/clinical_epidemiology/oxford.htm. Accessed February 27, 2020.

[19] Zhang J, Yu KF (1998). What’s the relative risk? A method of correcting the odds ratio in cohort studies of common outcomes. JAMA.

[20] Matusevicius M, Paciaroni M, Caso V, Bottai M, Khurana D, de Bastos M, et al. Outcome after intravenous thrombolysis in patients with acute lacunar stroke: an observational study based on SITS international registry and a meta-analysis. Int J Stroke 2019;0:1–9. 10.1177/1747493019840947 30935349

[21] Griebe M, Fischer E, Kablau M, Eisele P, Wolf ME, Chatzikonstantinou A, Gass A, Hennerici MG, Szabo K (2014). Thrombolysis in patients with lacunar stroke is safe: an observational study. J Neurol.

[22] Eggers CCJ, Bocksrucker C, Seyfang L (2017). The efficacy of thrombolysis in lacunar stroke – evidence from the Austrian stroke unit registry. Eur J Neurol.

[23] Hwang Y-H, Seo J-G, Lee H-W, Park S-P, Suh C-K (2008). Early neurological deterioration following intravenous recombinant tissue plasminogen activator therapy in patients with acute lacunar stroke. Cerebrovasc Dis.

[24] Lahoti S, Gokhale S, Caplan L, Michel P, Samson Y, Rosso C, Limaye K, Hinduja A, Singhal A, Ali S, Pettigrew LC, Kryscio R, Dedhia N, Hastak S, Liebeskind DS (2014). Thrombolysis in ischemic stroke without arterial occlusion at presentation. Stroke.

[25] Shobha N, Fang J, Hill MD (2013). Do lacunar strokes benefit from thrombolysis? Evidence from the registry of the Canadian stroke network. Int J Stroke.

[26] Paek YM, Lee JS, Park HK, Cho YJ, Bae HJ, Kim BJ, Park JM, Lee SJ, Cha JK, Park TH, Lee KB, Lee J, Lee BC, Kim JT, Kim DE, Shin DI, Kim WJ, Sohn SI, Choi JC, Hong KS (2019). Intravenous thrombolysis with tissue-plasminogen activator in small vessel occlusion. J Clin Neurosci.

[27] Yang L, Cao W, Wu F, Ling Y, Cheng X, Dong Q (2016). Predictors of clinical outcome in patients with acute perforating artery infarction. J Neurol Sci.

[28] Chang JJ, Chiem T, Alderazi YJ, Chapple K, Restrepo L (2013). Clinical outcomes after intravenous fibrinolysis in cryptogenic strokes with or without patent foramen ovale. J Stroke Cerebrovasc Dis.

[29] Sung S-F, Wu C-S, Hsu Y-C, Tseng M-C, Chen Y-W (2013). Oxfordshire community stroke project classification but not NIHSS predicts symptomatic intracerebral hemorrhage following thrombolysis. J Neurol Sci.

[30] Lee SJ, Saver JL, Liebeskind DS, Ali L, Ovbiagele B, Kim D (2011). Safety of intravenous fibrinolysis in imaging-confirmed single penetrator artery infarcts. Imaging.

[31] Fluri F, Hatz F, Rutgers MP, Georgiadis D, Sekoranja L, Schwegler G, Sarikaya H, Weder B, Müller F, Lüthy R, Arnold M, Reichhart M, Mattle HP, Tettenborn B, Nedeltchev K, Hungerbühler HJ, Sztajzel R, Baumgartner RW, Michel P, Lyrer PA, Engelter ST (2010). Intravenous thrombolysis in patients with stroke attributable to small artery occlusion. Eur J Neurol.

[32] Kohrmann M, Nowe T, Huttner HB, Engelhorn T, Struffert T, Kollmar R (2009). Safety and outcome after thrombolysis in stroke patients with mild symptoms. Cerebrovasc Dis.

[33] Padma MV, Singh MB, Bhatia R, Srivastava A, Tripathi M, Shukla G, Goyal V, Singh S, Prasad K, Behari M (2007). Hyperacute thrombolysis with IV rtPA of acute ischemic stroke: efficacy and safety profile of 54 patients at a tertiary referral center in a developing country. Neurol India.

[34] Cocho D, Belvis R, Marti-Fabregas J, Bravo Y, Aleu A, Pagonabarraga J (2006). Does thrombolysis benefit patients with lacunar syndrome?. Eur Neurol.

[35] Miedema I, Luijckx GJ, Brouns R, De Keyser J, Uyttenboogaart M (2016). Admission hyperglycemia and outcome after intravenous thrombolysis: is there a difference among the stroke-subtypes?. BMC Neurol.

[36] Simonsen CZ, Schmitz ML, Madsen MH, Mikkelsen IK, Chandra RV, Leslie-Mazwi T, Andersen G (2016). Early neurological deterioration after thrombolysis: clinical and imaging predictors. Int J Stroke.

[37] Pan Y-T, Lee J-D, Lin Y-H, Huang Y-C, Weng H-H, Lee M, Wu CY, Hsu HL, Yang HT, Hsu CY, Lee TH, Liu SJ, Peng TY, Liou CW, Chang KC, Huang YC (2016). Comparisons of outcomes in stroke subtypes after intravenous thrombolysis. Springerplus.

[38] Zivanovic Z, Gubi M, Vlahovic D, Milicevic M, Jovicevic M, Lucic A, et al. Patients with acute lacunar infarction have benefit from intravenous thrombolysis. J Stroke Cerebrovasc Dis 2018;1–6. 10.1016/j.jstrokecerebrovasdis.2018.10.020 30409747

[39] Kim DH, Lee DS, Nah HW, Cha JK (2018). Clinical and radiological factors associated with unfavorable outcome after intravenous thrombolysis in patients with mild ischemic stroke. BMC Neurol.

[40] Wardlaw JM, Smith EE, Biessels GJ, Cordonnier C, Fazekas F, Frayne R, Lindley RI, O'Brien JT, Barkhof F, Benavente OR, Black SE, Brayne C, Breteler M, Chabriat H, Decarli C, de Leeuw FE, Doubal F, Duering M, Fox NC, Greenberg S, Hachinski V, Kilimann I, Mok V, Oostenbrugge Rv, Pantoni L, Speck O, Stephan BC, Teipel S, Viswanathan A, Werring D, Chen C, Smith C, van Buchem M, Norrving B, Gorelick PB, Dichgans M, STandards for ReportIng Vascular changes on nEuroimaging (STRIVE v1) (2013). Neuroimaging standards for research into small vessel disease and its contribution to ageing and neurodegeneration. Lancet Neurol.

[41] Shi Y, Wardlaw JM (2016). Update on cerebral small vessel disease: a dynamic whole-brain disease. Stroke Vasc Neurol.

[42] Mustanoja S, Meretoja A, Putaala J, Viitanen V, Curtze S, Atula S, Artto V, Häppölä O, Kaste M, for the Helsinki Stroke Thrombolysis Registry Group (2011). Outcome by stroke etiology in patients receiving thrombolytic treatment: descriptive subtype analysis. Stroke.

[43] OCEBM Levels of Evidence Working Group. The Oxford 2011 Levels of Evidence Oxford Centre for Evidence-Based Medicine 2011. https://www.cebm.net/wp-content/uploads/2014/06/CEBM-Levels-of-Evidence-2.1.pdf. Accessed 15 Nov 2020.

[44] Bamford J, Sandercock P, Jones L, Warlow C (1987). The natural history of lacunar infarction: the oxfordshire community stroke project. Stroke.

[45] Boiten J, Lodder J (1993). Prognosis for survival, handicap and recurrence of stroke in lacunar and superficial infarction. Cerebrovasc Dis.

[46] Khatri P, Kleindorfer DO, Devlin T, Sawyer RN, Starr M, Mejilla J (2018). Effect of alteplase vs aspirin on functional outcome for patients with acute ischemic stroke and minor nondisabling neurologic deficits the PRISMS randomized clinical trial. JAMA.

[47] Samuelsson M, Söderfeldt B, Olsson GB (1996). Functional outcome in patients with lacunar infraction. Stroke.

[48] Sacco SE, Whisnant JP, Broderick JP, Phillips SJ, Michael WOF (1991). Epidemiological characteristics of lacunar infarcts in a population. Stroke.

[49] Burgess S (2016). Estimating and contextualizing the attenuation of odds ratios due to non-collapsibility. Communications in Statistics - Theory and Methods.

[50] Mead GE, Lewis SD, Wardlaw JM, Dennis MS, Warlow CP (2000). How well does the Oxfordshire community stroke project classification predict the site and size of the infarct on brain imaging?. J Neurol Neurosurg Psychiatry.

