# Supplementary material for: Efficacy and Safety of Intravenous rtPA in Ischemic Strokes Due to Small-Vessel Occlusion: Systematic Review and Meta-Analysis
Source: Transl Stroke Res. 2021 Feb 28;12(3):406–15. doi: 10.1007/s12975-021-00890-9 (PMC8055574; doi:10.1007/s12975-021-00890-9)
Supplement: Supplementary file 1 — (DOCX 1.44 MB) [file 12975_2021_890_MOESM1_ESM.docx]

**Title: Efficacy and safety of intravenous rtPA in ischemic strokes due to small-vessel occlusion: systematic review and meta-analysis**

Bartosz Karaszewski (0000-0003-2726-0016)^1,2,3^, Prof., MD, PhD; Adam Wyszomirski (0000-0002-6293-1439)^1^, MSc; Bartosz Jabłoński (0000-0002-1657-5090)^1,2^, MD; David J Werring (0000-0003-2074-1861)^4^, Prof., PhD; Dominika Tomaka^2^, MD

^1^Department of Adult Neurology, Division of Neurology, Faculty of Medicine, Medical University of Gdansk, Gdansk, Poland

^2^Department of Adult Neurology, University Clinical Center in Gdansk, Gdansk, Poland

^3^Prof. Karaszewski serves as the Main Expert in Stroke Medicine for the Polish Ministry of Health

^4^Stroke Research Centre, University College London, Queen Square Institute of Neurology, London, United Kingdom

Correspondence: Prof. Bartosz Karaszewski; MD, PhD, Department of Adult Neurology, Medical University of Gdansk, University Clinical Center in Gdansk, Debinki 7, 80-211 Gdansk. Email [bartosz@karaszewski.org](mailto:bartosz@karaszewski.org), Tel. +48 58 349 23 00, Fax +48 58 349 23 20

**SUPPLEMENTAL MATERIAL**

**Supplemental Table I.** SVD/LC and sICH definitions used in the analyzed studies

| **Study** | **Neuroimaging/clinical criteria** | **sICH def.** |
| --- | --- | --- |
| Griebe et al. | ASCO class. MRI (DWI-confirmed) | Significant neurological deterioration ≥ 4 points NIHSS |
| Eggers et al | OCSP, TOAST, MRI | NINDS criteria |
| Hwang et al. | OCSP, TOAST, MRI | Early neurological deterioration (END) |
| Lahoti et al. | OCSP, TOAST, MRI | Significant neurological deterioration ≥ 4 points NIHSS |
| NINDS | OCSP, TOAST, CT | NINDS criteria |
| Shobha et al. | OCSP | ECASS; deterioration ≥ 4 points NIHSS |
| Lindley et al. | OCSP + MRI/CT, TOAST | Significant neurological deterioration |
| Barow et al. | DWI-FLAIR mismatch | Safe Implementation of Thrombolysis in Stroke–Monitoring Study (SITS-MOST) criteria |
| Peak et al. | TOAST, MRI + MRA/CTA | ECASS-3 criteria |
| Yang et al. | TOAST, MRI/CT; OCSP - for patients with negative findings on brain imaging | not specified |
| Matusevicius et al. | TOAST, CT/MRI | SITS-MOST, NINDS, ECASS-2 criteria |
| Chang et al. | TOAST, MRI/CT/CTA | not specified |
| Sung et al. | OCSP | Significant neurological deterioration ≥ 4 points NIHSS |
| Lee et al. | OCSP | SITS-MOST criteria |
| Fluri et al. | TOAST, MRI/CT - SAO | NINDS criteria |
| Kohrmann et al. | TOAST | NINDS criteria |
| Padma et al. | TOAST | not specified |
| Cocho et al. | OCSP, TOAST | ECASS-2 criteria |
| Miedema et al. | OCSP, CT | SITS-MOST criteria |
| Simonsen et al. | TOAST, MRI | SITS-MOST criteria |
| Pan et al. | TOAST | ECASS-2 criteria |
| Zivanovic et al. | OCSP, CT | ECASS-3 criteria |
| Kim et al. | TOAST, MRI/MRA | Significant neurological deterioration ≥ 4 points NIHSS |

**Supplemental Table II**. The quality assessment of individual trials comparing iv rtPA and control with Cochrane Collaboration’s tool [1]

| Study | Random sequence generation (selection bias) | Allocation concealment (selection bias) | Blinding of participants and personnel (performance bias) | Blinding of outcome assessment  (detection bias) | Incomplete outcome data (attribution bias) | Selective reporting (reporting bias) | Comments |
| --- | --- | --- | --- | --- | --- | --- | --- |
| NINDS group | Low risk | Low risk | Low risk | Low risk | Unclear risk | Low risk | Low risk |
| Lindley et al. | Low risk | Low risk | High risk | Low risk | Low risk | Low risk | Low risk |
| Barow et al. | Low risk | Low risk | Low risk | Low risk | Low risk | Low risk | Low risk |

**Supplemental Table III.** The quality assessment of individual observational comparative cohort studies with the Newcastle-Ottawa Scale [2]

| Study | Selection | | | | Comparability | | Outcome | | | Total score |
| --- | --- | --- | --- | --- | --- | --- | --- | --- | --- | --- |
|  | Representativeness of exposed cohort? | Selection of the non-exposed cohort? | Ascertainment of exposure? | Demonstration that outcome of interest was not present at start of study? | Study controls for age/sex? | Study controls for at least 3 additional risk factors? | Assessment of outcome? | Was follow-up long enough for outcome to occur? | Adequacy of follow-up of cohorts? |  |
| Griebe et al. | 1 | 1 | 1 | 0 | 1 | 0 | 1 | 1 | 1 | 7/9 |
| Eggers et al. | 1 | 1 | 1 | 1 | 1 | 1 | 1 | 1 | 1 | 9/9 |
| Hwang et.al | 1 | 1 | 1 | 0 | 1 | 0 | 1 | 1 | 1 | 7/9 |
| Lahoti et al. | 1 | 1 | 1 | 1 | 0 | 0 | 1 | 1 | 1 | 7/9 |
| Shobha et al. | 1 | 1 | 1 | 0 | 1 | 1 | 1 | 1 | 1 | 8/9 |
| Paek et al. | 1 | 1 | 1 | 1 | 1 | 1 | 1 | 1 | 1 | 9/9 |
| Yang et al. | 1 | 1 | 1 | 0 | 1 | 0 | 1 | 1 | 1 | 7/9 |
| Matusevicius et al. | 1 | 1 | 1 | 0 | 1 | 1 | 1 | 1 | 1 | 8/9 |

**Supplemental Table IV**. Adjustment for covariates

| Study | Adjustment for covariates |
| --- | --- |
| Lahoti et al. | Age, sex, NIHSS at presentation, hypertension, diabetes mellitus, hyperlipidemia, atrial fibrillation, and smoking. |
| Barow et al. | Age, symptom severity (NIHSS score). |
| Paek et al. | Age, sex, initial NIHSS, pre-stroke mRS score, coronary heart disease, initial systolic BP, pre-stroke antiplatelet, pre-stroke statin, onset to arrival time, and antithrombotic management after admission. |
| Matusevicius et al. | Age, sex, NIHSS at baseline, onset-to-door time, history of hypertension, diabetes, hyperlipidemia, smoking, previous stroke, atrial fibrillation, and congestive heart failure. |

**Supplemental Figure I.** PRISMA [3] flow diagram for the meta-analysis

Records excluded (n=**95**)

Records identified through databases searching (n=**229**)

Identification

Records excluded (n=**95**)

Records screened (n=**137**)

Screening

Full-text articles excluded (n=**19**)

Full-text articles assessed for eligibility (n=**42**)

Eligibility

Included

Studies included in quantitative synthesis (n=**23**)

**Supplemental Figure II.** Funnel plots of unadjusted analysis for a) excellent outcome; b) favorable outcome; c) incidence of sICH in rtPA vs control patients; d) sICH in rtPA patients


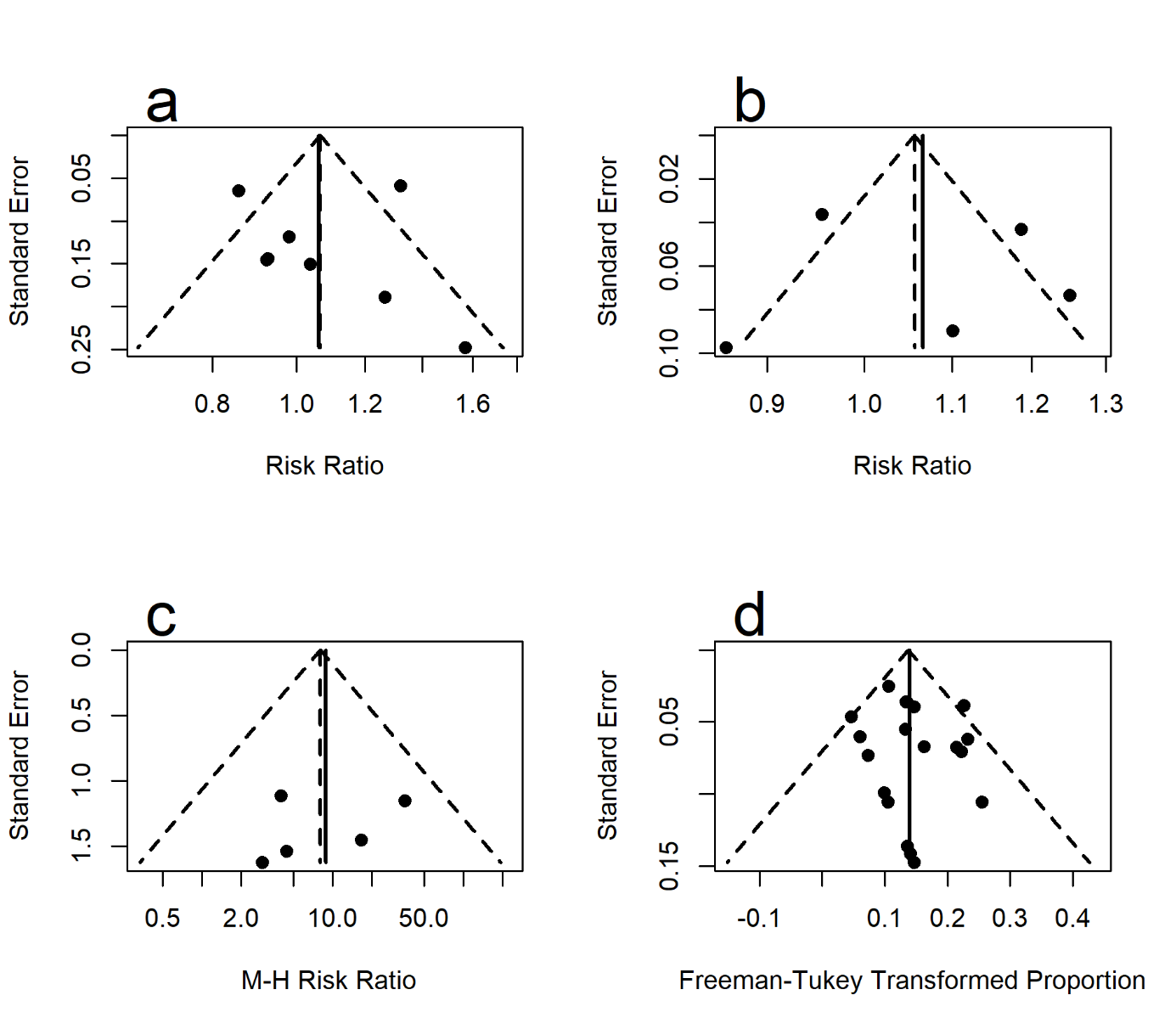


**Supplemental Figure III.** The unadjusted pooled risk ratio for excellent functional outcome


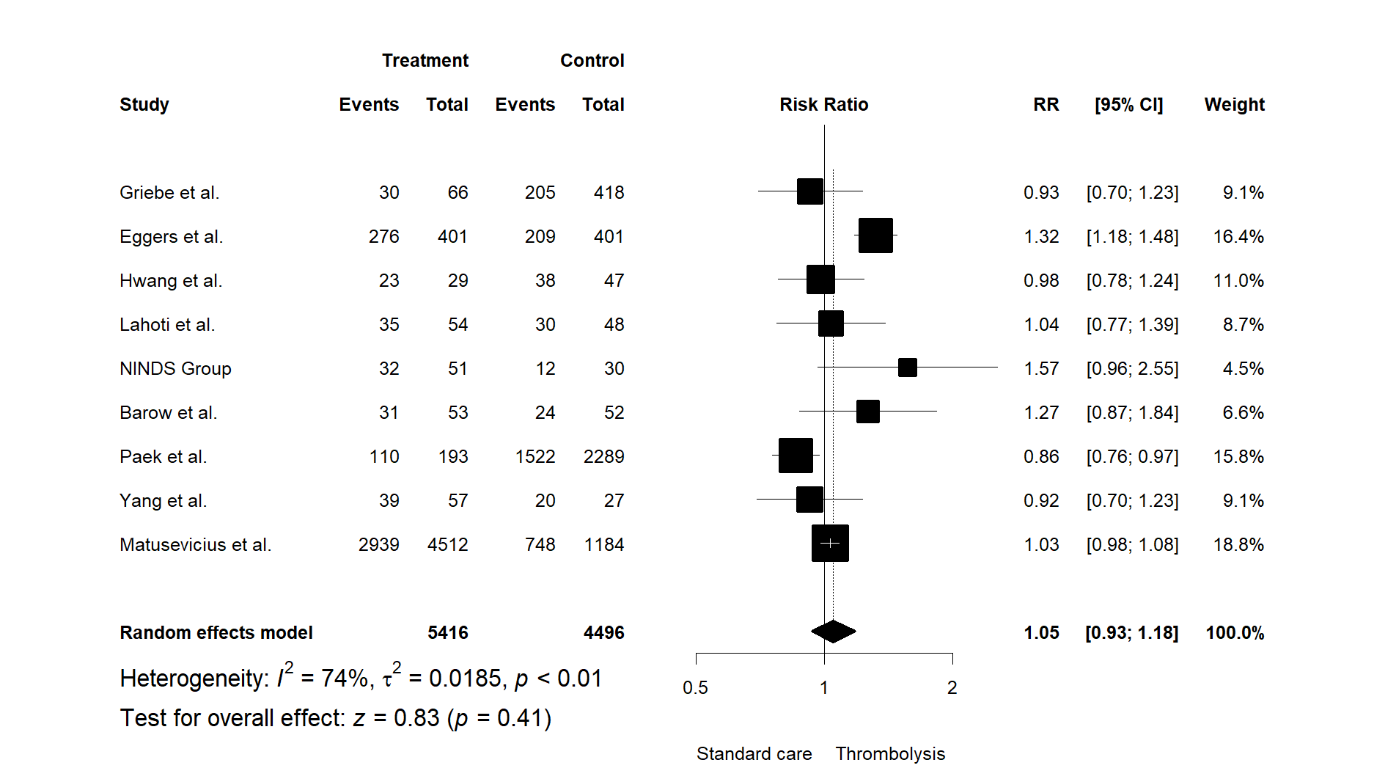


**Supplemental Figure IV.** The unadjusted overall risk ratio for favorable functional outcome


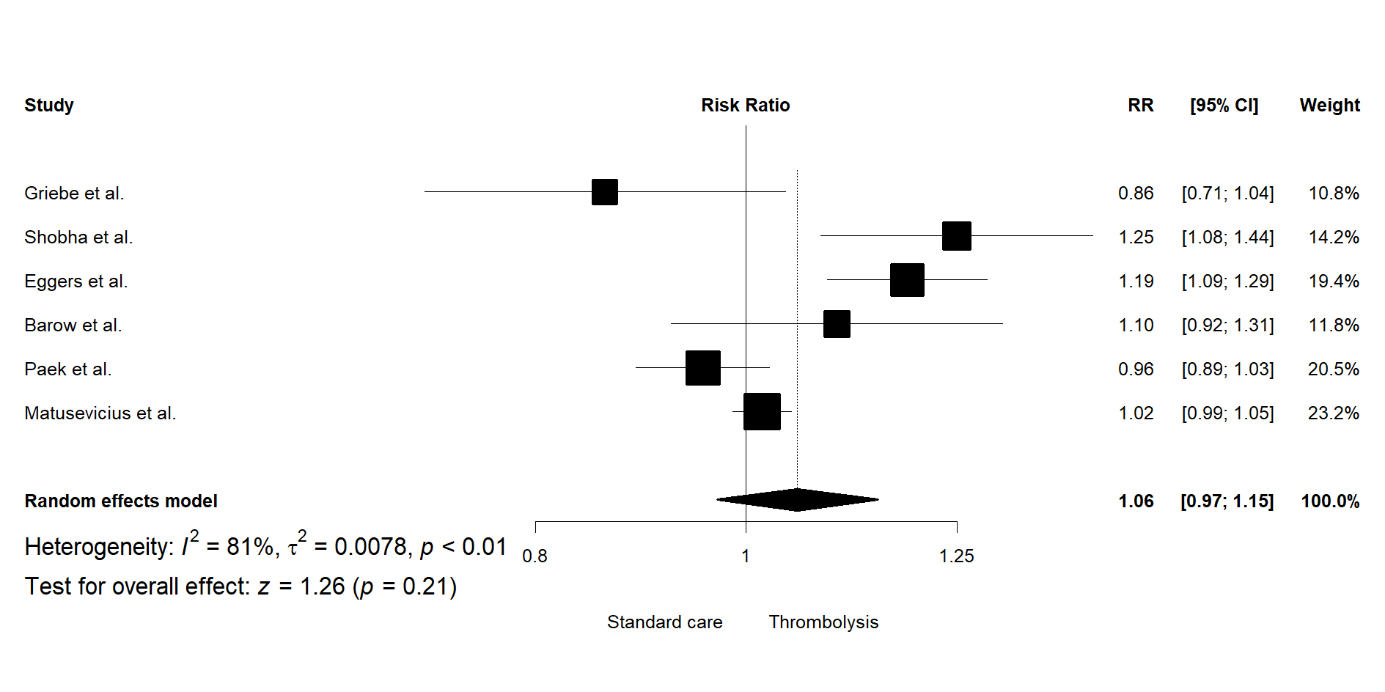


**Supplemental Figure V.** The unadjusted pooled risk ratio for excellent functional outcome excluding the SITS cohort
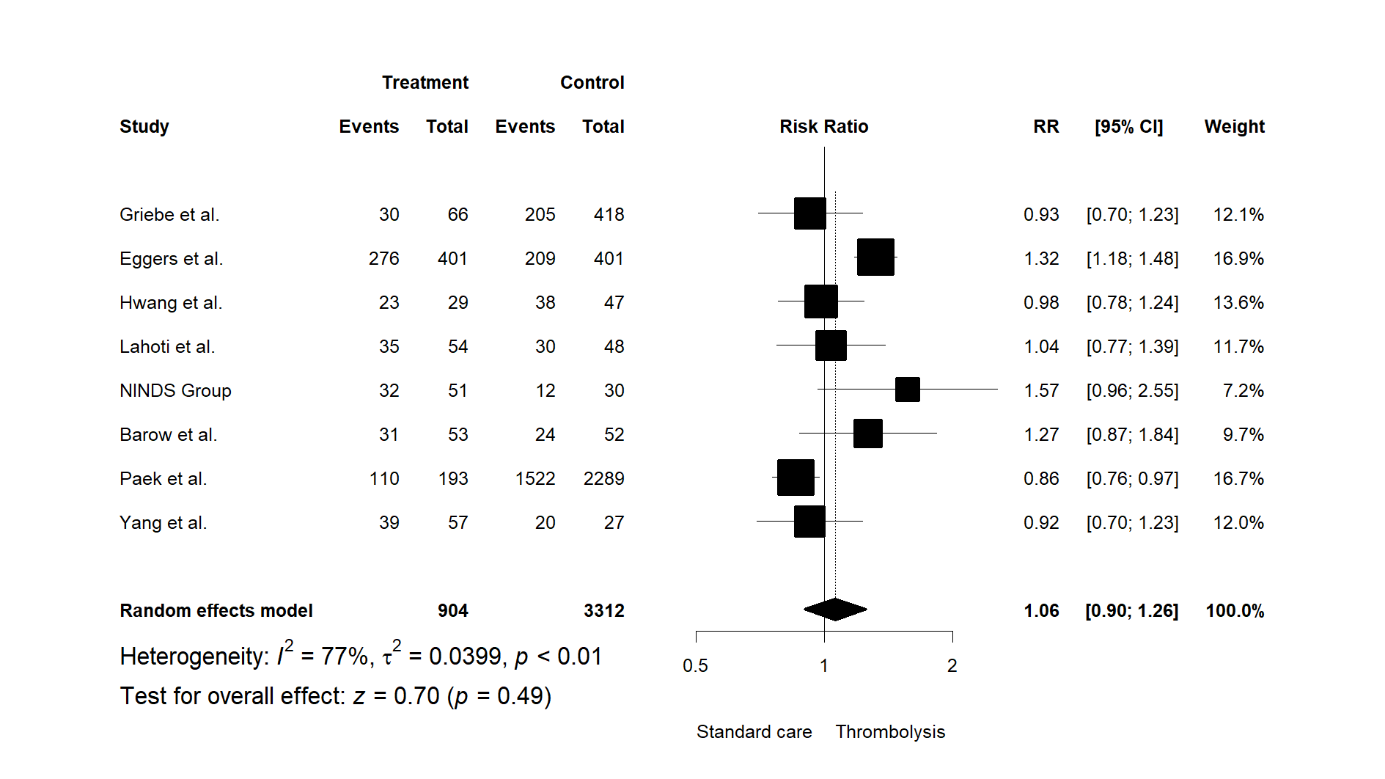


**Supplemental Figure VI.** The unadjusted overall risk ratio for favorable functional outcome excluding the SITS cohort
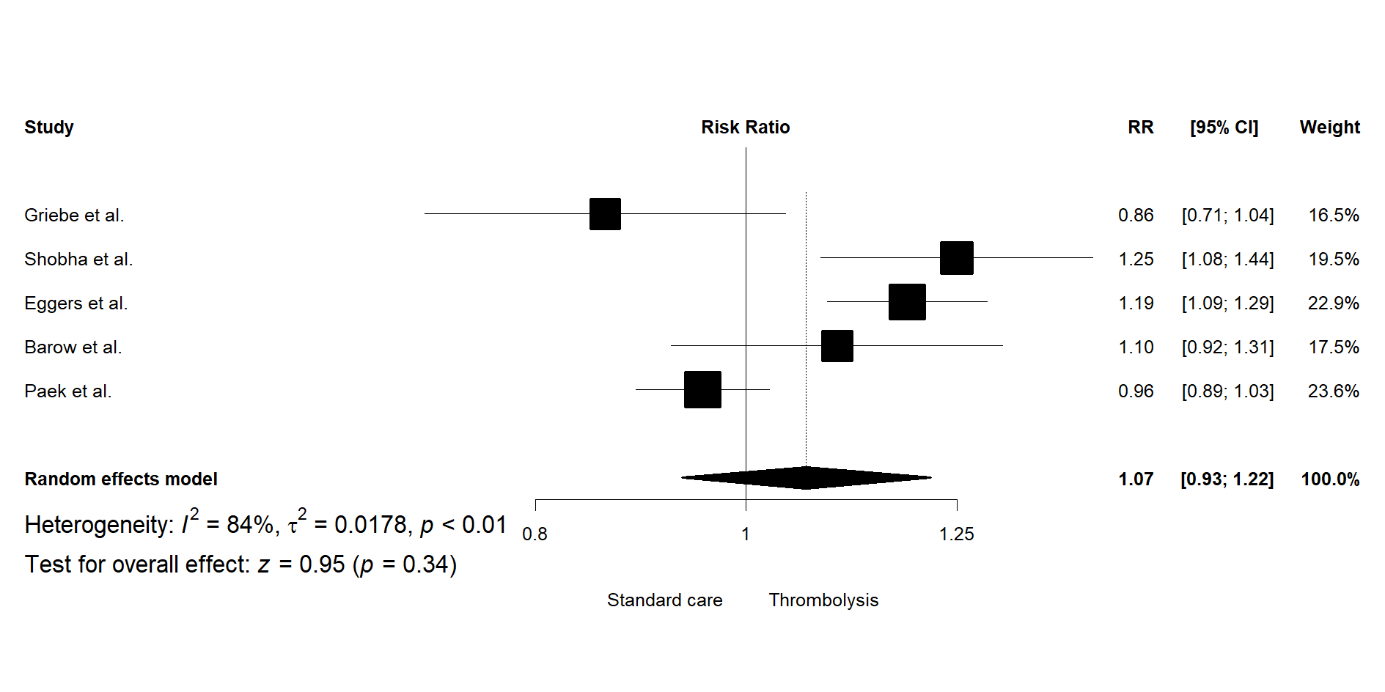


**Supplemental Figure VII.** The adjusted pooled odds ratio for excellent functional outcome excluding the SITS cohort


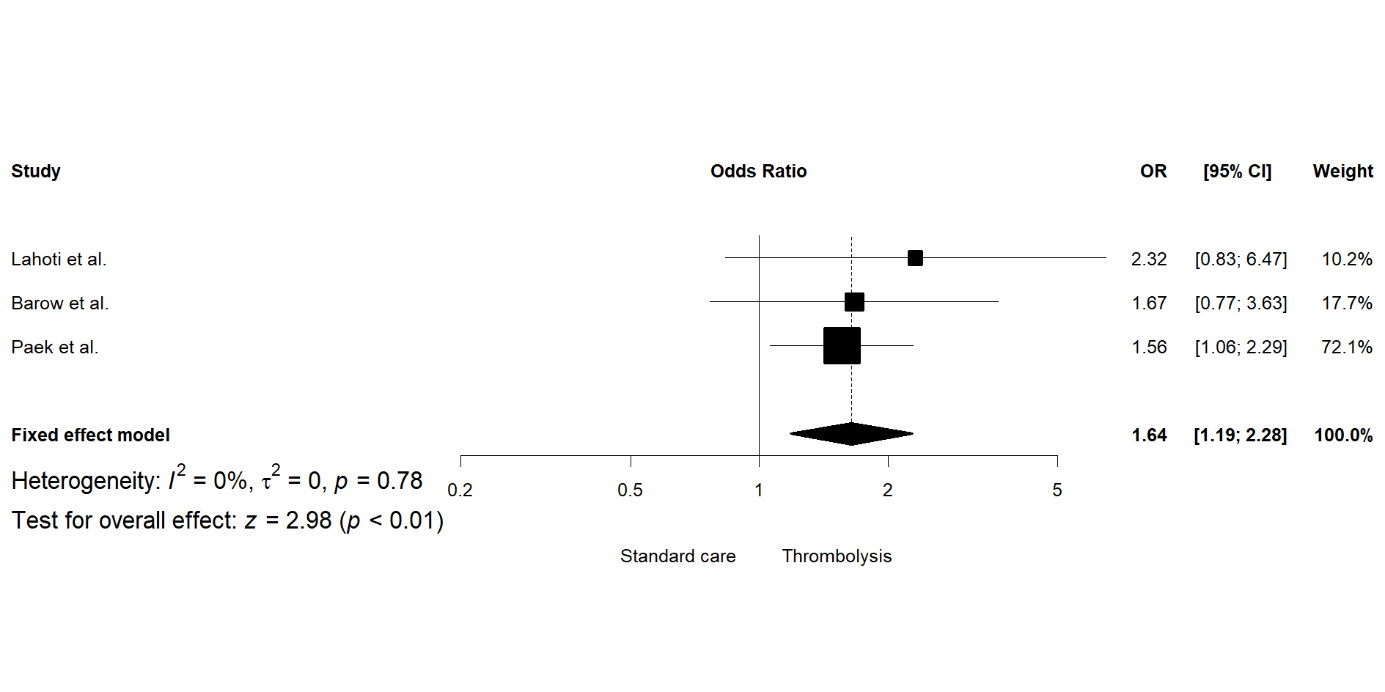


**Supplemental Figure VIII.** The pooled rate of sICH in trombolysed patients excluding the SITS cohort


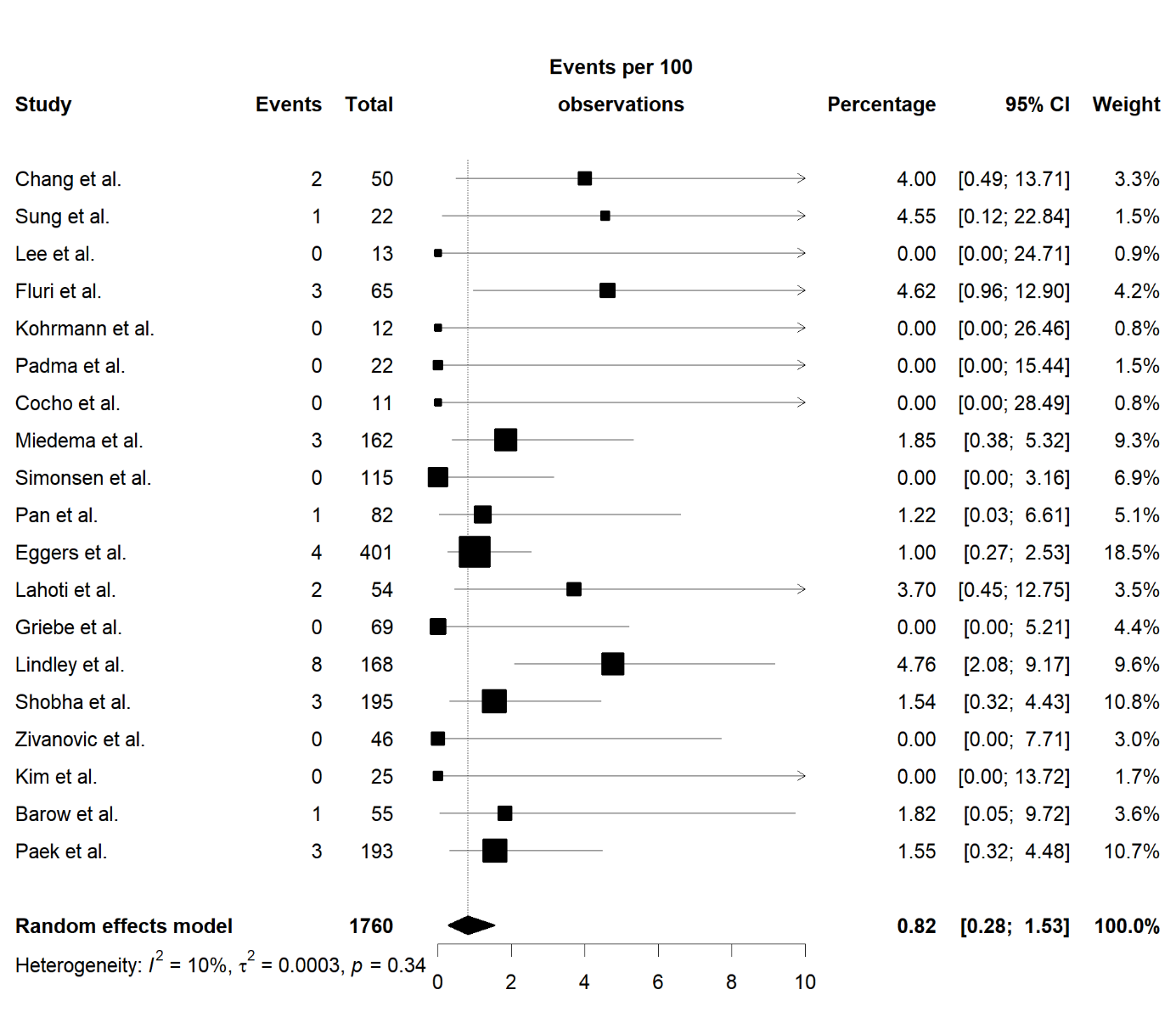


**References**

1. Higgins JPT, Altman DG, Gotzsche PC, Juni P, Moher D, Oxman AD, et al. The Cochrane Collaboration’s tool for assessing risk of bias in randomised trials. *BMJ*. 2011;343:d5928.

2. Wells Ga, Shea B, O’Connell D, Peterson J, Welch V, Losos M, et al. The Newcastle-Ottawa Scale (NOS) for assessing the quality if nonrandomized studies in meta-analyses. http://www.ohri.ca/programs/clinical_epidemiology/oxford.htm. Accessed February 27, 2020.

3. Liberati A, Altman DG, Tetzlaff J, Mulrow C, Gøtzsche PC, Ioannidis JPA, et al. The PRISMA statement for reporting systematic reviews and meta-analyses of studies that evaluate health care interventions: explanation and elaboration. *J. Clin. Epidemiol.* 2009;62:e1-34.
